# Factors Associated With Morbidity and Retreatment After Surgical Management of Nonacute Subdural Hematomas in Elderly Patients

**DOI:** 10.7759/cureus.24779

**Published:** 2022-05-06

**Authors:** Michael T Bounajem, Geoffrey Peitz, Roman Fernandez, Zhu Wang, Michael McGinity, Ramesh Grandhi

**Affiliations:** 1 Neurosurgery, University of Utah, Salt Lake City, USA; 2 Neurosurgery, University of Texas Health Science Center at San Antonio, San Antonio, USA; 3 Population Health Sciences, University of Texas Health Science Center at San Antonio, San Antonio, USA

**Keywords:** craniotomy, burr hole, statins, recurrence, mixed density, subacute, chronic, subdural hematoma

## Abstract

Background

Preoperative identification of clinical, radiographic, and surgery-specific factors associated with nonacute subdural hematomas (SDHs) may enable clinicians to optimize the efficacy of the initial surgical intervention, improve outcomes, and decrease rates of surgical recurrence.

Methods

The authors identified patients aged ≥65 years who underwent surgical treatment of chronic, subacute, or mixed-density SDH at a level-1 trauma hospital over a ten-year period (2010-2019). Pre-and postoperative clinical, radiographic, and surgery-specific data were collected. Predictors of surgical recurrence as well as morbidity, mortality, and discharge disposition were analyzed.

Results

There were 268 nonacute SDHs treated surgically; 46 were chronic, 19 were subacute, and 203 were mixed density. Of these, 179 were treated with burr hole(s), 62 with miniature craniotomy, and 27 via a large craniotomy and removal of subdural membranes. Statin use was protective (OR 0.22; 95% CI 0.08, 0.60) against recurrence requiring reoperation. Preoperative use of antithrombotic agents was not significantly associated with increased recurrence requiring reoperation. Smaller preoperative hematoma thickness was associated with significantly lower mortality risk, whereas mixed-density hematomas, patient age, change in thickness after surgery, density, and presence of cisternal effacement were significantly associated with discharge disposition. Hematoma type was also associated with hospital and intensive care length of stay.

Conclusions

Our experience suggests that, in elderly patients, premorbid statin usage is associated with lower recurrence rates and preoperative antithrombotic use does not affect recurrence when appropriately reversed before surgery. Patient age, preoperative thickness, and hematoma type contribute to postoperative outcomes such as discharge disposition and length of stay.

## Introduction

Subdural hematomas (SDHs) are a common pathology encountered by neurosurgeons and neurocritical care specialists. The aggregate medical charges for patients with SDHs were nearly $2 billion in the United States in 2006, with more recent reports of individual patient 1-year cost after surgical evacuation of $72,799, reflecting the significant economic burden on the healthcare system [1,2]. Most SDHs are nonacute (chronic, subacute, mixed density); the annual incidence of chronic SDHs is approximately 13.3 per 100,000 in the general population, with approximately two-thirds of cases occurring in patients 65 years or older [1,3]. Furthermore, there have been substantial increases in incidence in recent decades [4]. The cause of the increasing number of SDHs encountered in clinical practice is multifactorial, involving, among other factors, the aging of the population, increased susceptibility to injury due to comorbid conditions and use of antithrombotic agents, improved access to imaging and diagnosis, and a growing epidemic of falls among the elderly population. Safe, effective, and durable treatment of nonacute SDHs is essential.

Both surgical and nonsurgical strategies have been employed for the management of nonacute SDHs. Although burr-hole drainage is the most common method of surgical evacuation, no consensus has been reached on which method is most appropriate [5,6]. Given the pathophysiology and common comorbidities among elderly patients with nonacute SDHs, surgical recurrence requiring reoperation is particularly troublesome. Recurrence rates range from 5 to 33%, with most instances occurring 1-8 weeks postoperatively [3,5]. Many factors have been implicated in the recurrence after initial surgical intervention, including older age, anticoagulant/antiplatelet drug usage, postoperative pneumocephalus, midline shift, brain atrophy, the thickness of inner hematoma membranes, and a variety of metrics for the size and amount of hematoma evacuated [7-9]. The association of these factors with recurrence, however, has been very inconsistently demonstrated, with many studies reporting conflicting results. We examined preoperative and surgery-related factors, including volumetric analysis and the impact of statins, that may be associated with recurrence and poor outcomes in elderly patients with nonacute SDHs.

## Materials and methods

We retrospectively reviewed the records of all patients 65 years and older who presented with chronic, subacute, or mixed-density SDHs who underwent surgical treatment over a 10-year period (2010-2019) at the University of Texas Health San Antonio University Hospital, a level-1 trauma center. The institutional review board of the University of Texas Health San Antonio (IRB protocol 15-0808H) approved this study with a waiver of informed consent. All patient information was de-identified and analyzed in compliance with Health Insurance Portability and Accountability Act regulations.

**Figure 1:**
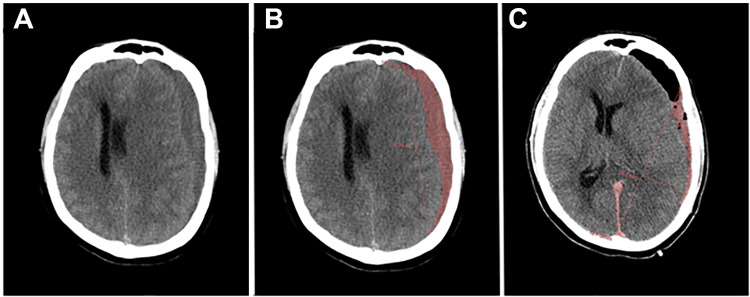
Axial CT images of a patient with chronic SDH (A) Preoperative and (B) preoperative with hematoma volume highlighted in red using Philips Multi-Modality Tumor Tracking software. (C) Postoperative with hematoma volume highlighted.

Baseline demographic and clinical data including age, sex, presenting Glasgow Coma Scale (GCS) score, and antithrombotic/steroid/statin usage, as well as surgery-specific factors, including the type of craniotomy (i.e., burr-hole(s), miniature craniotomy <20 cm^3^, or large craniotomy >20 cm^3^) and placement of subdural/subgaleal drains or both, were collected. Patients were considered to be antithrombotic users if an antithrombotic medication was indicated as a home medication upon admission and as a statin-user if statins were prescribed as a component of posthospitalization discharge orders. Surgical decision-making was at the discretion of the attending neurosurgeon. Computed tomography (CT) scans were reviewed to identify the nonacute SDH subtype, hematoma density in Hounsfield units, maximal pre-and postoperative axial hematoma thickness, presence of midline shift, cisternal effacement, presence of loculations, and laterality of hematoma location. Pre-and postoperative hematoma volume was assessed using Multimodality Tumor Tracking software (Philips, Amsterdam, Netherlands) (Figure 1). Maximal hematoma thickness and volume were measured on the final CT scan before discharge. Subsequent calculations included postoperative percentage change in maximal hematoma thickness ({preoperative thickness - postoperative thickness}/preoperative thickness) and postoperative percentage change in hematoma volume ({preoperative volume - postoperative volume}/preoperative volume). Radiographic factors were also scored with the Oslo CSDH grading system and compared with actual rates of recurrence [10].

The primary outcome was SDH recurrence requiring reoperation; secondary outcomes included total length of stay (LOS), intensive care unit (ICU) LOS, complication rate and type, discharge disposition (home, inpatient rehabilitation facility, skilled nursing facility, hospice, or death), and mortality. Univariate and multivariate logistic regression analysis and computation of odds ratios with 95% confidence intervals were conducted for binary outcomes such as recurrence. A p-value of <0.05 was used as a cutoff to determine a significant difference. Bivariate and multivariate logistic regression analysis was conducted for continuous outcomes such as LOS and ICU LOS. Although we attempted to minimize the number of variables for analysis to prevent overfitting, perfect adherence to the one-in-ten rule of thumb was not achieved given the important preoperative factors and outcomes that needed to be assessed.

## Results

During the 10-year period studied, surgical intervention was performed for 268 nonacute SDHs in 244 patients, of which 46 were chronic, 19 were subacute, and 203 were mixed density (Table 1). The average age of the patients was 75.9±6.72 years, and the mean GCS score at presentation was 13±2.2. Eighty-one patients were female and 163 were male. On head CT, 100 hematomas were loculated, 154 patients had midline shift, and 114 had cisternal effacement. There were 220 patients with unilateral hematomas and 24 with bilateral hematomas requiring surgical intervention. Eighty-nine patients were taking a statin at the time of presentation, 94 were taking antiplatelet agents, and 53 were taking anticoagulant medications. Of note, all surgically treated patients underwent reversal of antithrombotic medication; those on antiplatelet agents received intravenous desmopressin or platelets, whereas those on anticoagulant agents received vitamin K and fresh frozen plasma or 4-factor prothrombin complex concentrate. Twenty-seven hematomas were evacuated via a large craniotomy, 62 with a miniature craniotomy, and 179 with burr-hole(s) drainage. Drains were placed in 183 cases, of which 130 were subdural, 50 subgaleal, and three epidural. The placement of drains was at the discretion of the attending neurosurgeon.

**Table 1:** Preoperative clinical information for patients with nonacute subdural hematomas, subdivided based on eventual recurrence requiring reoperation SDH, subdural hematoma; GCS, Glasgow Coma Scale. Values are reported as mean (±SD) or number (%)

Variable	All	Recurrent	Nonrecurrent	p-value	
				Univariate analysis	Multivariate analysis
Number of cases	268	41 (15.3)	227 (84.7)		
Mean age (years)	75.9±6.72	75.6±6.7	76.1±6.89	0.65	0.68
Sex			146/81	0.1	0.07
Male	178 (66.4)	32 (11.9)	146 (54.5)		
Female	90 (33.6)	9 (3.36)	81 (30.2)		
SDH type				0.44	0.6
Chronic	46 (17.2)	8 (2.99)	38 (14.2)		
Subacute	19 (7.09)	4 (1.49)	15 (5.59)		
Mixed density	203 (75.8)	29 (10.8)	174 (64.9)		
Mean presenting GCS	13.3±2.2	13.6±1.66	13.2±2.28	0.269	0.37
Anticoagulant usage				0.64	0.67
Yes	53 (19.8)	7 (2.61)	46 (17.2)		
No	215 (80.2)	34 (12.7)	181 (67.5)		
Antiplatelet usage				0.74	0.27
Yes	94 (35.1)	16 (5.97)	78 (29.1)		
No	174 (64.9)	25 (9.33)	149 (55.6)		
Statin usage				0.004	0.004
Yes	89 (33.2)	5 (1.87)	84 (31.3)		
No	179 (66.8)	36 (13.4)	143 (53.4)		
Steroid usage				0.48	0.77
Yes	28 (10.5)	3 (1.12)	25 (9.33)		
No	240 (89.6)	38 (14.2)	202 (75.4)		
Cisternal effacement	114 (42.5)	16 (5.97)	98 (36.6)	0.62	0.67
Midline shift	154 (57.5)	25 (9.33)	129 (48.1)	0.62	0.34
Surgical treatment				0.53	0.97
Burr hole	179 (66.8)	27 (10.1)	152 (56.7)		
Miniature craniotomy	62 (23.1)	7 (2.61)	55 (20.5)		
Full craniotomy	27 (10.1)	7 (2.61)	20 (7.46)		

A total of 41 hematomas (15.3%) recurred and required reoperation after the index operation. Reoperation rates among patients on anticoagulant agents (7 of 46, 15.2%) and antiplatelets (16 of 78, 20.5%) were not significantly different compared with reoperation rates for patients not taking such medications (p=0.67, p=0.27, respectively) (Table 1). The average initial preoperative hematoma thickness among patients who eventually required reoperation was not significantly different from those who did not (22.5 mm vs. 20.8 mm, p=0.08) (Table 2). In addition, the initial preoperative volume was similar in both groups (139 mL in the recurrent group vs. 137 mL in the nonrecurrent, p=0.52). The percent change in hematoma thickness after initial surgery (60.4% vs. 61.4%) was not different in patients who required reoperation and those who did not (p=0.59), nor was the percent change in volume (68.2% vs. 71.2%, p=0.24). Only five of the 89 (5.9%) hematomas in patients on statins required reoperation, a rate that was significantly lower on both univariate and multivariate analysis (OR 0.22; 95% CI 0.08, 0.60, p=0.004).

**Table 2:** Comparison of radiographic findings in recurrent and nonrecurrent SDHs Values are reported as mean (±SD).

Variable	All	Recurrent	Nonrecurrent	p-value	
				Univariate analysis	Multivariate analysis
Preoperative thickness (mm)	21±6.41	22.5±7.53	20.8±6.17	0.12	0.08
Postoperative thickness (mm)	8.17±5.01	9.1±6.66	8±4.64		
% change in thickness	61.2±19.8	60.4±24.3	61.4±18.9	0.76	0.59
Preoperative volume (ml)	137±56.3	139±56.1	137±56.4	0.88	0.52
Postoperative volume (ml)	41.8±37.4	44.8±42.7	41.3±36.4		
% change in volume	70.8±19.9	68.2±26.1	71.2±18.6	0.37	0.24
Hematoma density (Hounsfeld units)	36.9±11.9	35.2±11.9	37.2±11.9	0.32	0.39

Secondary outcomes recorded included LOS, ICU LOS, discharge disposition, complications, and mortality (Table 3). The hematoma subtype was associated with significantly different LOS and ICU LOS on univariate analysis (p<0.001) and multivariate analysis (p<0.001). Discharge disposition consisted of five categories: home, skilled nursing facility, long-term acute care facility, inpatient rehabilitation, and death. In total, 120 cases were discharged home, 62 to a skilled nursing facility, 49 to a long-term acute care facility, seven to inpatient rehabilitation, and six died in the hospital. At the latest follow-up (six months when available), the total number of deaths was 16, with 15 (93.8%) among patients with mixed-density hematomas. Factors associated with discharge disposition included cisternal effacement (p<0.001), hematoma density (p=0.04), change in thickness of SDH after initial surgical intervention (p=0.007), age (p=0.02), hematoma subtype (p<0.001), and presenting GCS (p=0.04) on multivariate analysis. The overall complication rate was 10.8%, comprising nine cases of pneumonia, eight cases of deep vein thrombosis, two cases of bacteremia, and 12 cases of urinary tract infections. Preoperative SDH thickness was the only factor associated with significantly greater mortality on multivariate analysis (p=0.04).

**Table 3:** Outcomes data for patients with chronic, subacute, and mixed-density hematomas DVT, deep vein thrombosis; UTI, urinary tract infection; SNF, skilled nursing facility; LTAC, long-term acute care; GCS, Glasgow Coma Scale

Variable	All (N=268)	Significant predictors (univariate analysis)	Significant predictors (multivariate analysis)
Length of stay (days)			
Total hospital	7.01±4.69	hematoma subtype (p<0.001)	hematoma subtype (p<0.001)
Intensive care unit	3.75±3.66	hematoma subtype (p<0.001); GCS (p=0.006)	hematoma subtype (p<0.001)
Complication	27 (10.8%)		change in volume (p=0.04)
pneumonia	9		
DVT	8		
bacteremia	2		
UTI	12		
Discharge disposition (number of patients = 244)		cisternal effacement (p<0.001); change in thickness (p=0.002); density (p=0.01); hematoma subtype (p<0.001); GCS (p<0.001)	cisternal effacement (p<0.001); change in thickness (p=0.007); density (p=0.04); hematoma subtype (p<0.001); GCS (p=0.04); age (p=0.02)
Home	120 (49.2%)		
SNF	62 (25.4%)		
LTAC	49 (20.1%)		
Inpatient rehabilitation	7 (2.87%)		
In-hospital mortality	6 (2.46%)		
Mortality (6 month)	16 (6.56%)	preoperative thickness (p=0.005); change in thickness (p=0.01); change in volume (p=0.04); density (p=0.006); GCS (p=0.002)	preoperative thickness (p=0.04)

## Discussion

The incidence of nonacute SDH is expected to increase as the elderly proportion of the population increases [1]. Thus, efficient and effective management of this condition is essential, especially with regard to the prevention of recurrence requiring reoperation. Although many studies have reported factors that appear to be related to recurrence, the consistency of these factors has not been demonstrated in the extant literature. The cohort examined in this study had similar demographic characteristics to previously reported cohorts, with a mean age of 75.9 years and a male-to-female predominance of 2:1. The overall rate of recurrence was 15.3%, which is within the range of 5-33% commonly cited [3,5]. The average Oslo CSDH grade, a score that uses radiographic factors to predict the likelihood of recurrence on a scale of 1-5, was found to be 2 [10]. This corresponds to a rate of recurrence requiring reoperation of 6% (95% CI 1, 16). The mortality rate in our cohort was 6.56%, which is comparable with recently reported mortality rates of 4-18% [11]. Treatment strategies were also consistent with previous studies, with burr-hole craniotomy being the most common method of drainage employed.

Of the patients that required reoperation because of SDH recurrence, most initially presented with a mixed-density hematoma (70.7%), followed by chronic (19.5%), and finally subacute (9.76%). This is consistent with previous studies, which have shown higher rates of recurrence in mixed-density SDHs than in subacute or chronic SDHs [12,13]. Patients with mixed-density SDHs have also been found to have a greater propensity for postoperative seizures [14]. In mixed-density SDHs, an extremely fragile neomembrane forms, and local inflammatory mediators predispose the neomembrane to repeated bleeding [5,15,16]. Further disruption of this neomembrane, as well as increased intramembranous volume due to acute bleeding, may contribute to the greater rates of recurrence in mixed-density hematomas.

Statin usage has often been explored as a protective factor in various intracranial vascular pathologies. It has been reported that atorvastatin in particular increases the amount of circulating endothelial progenitor cells, thereby promoting angiogenesis [17]. Statins have also been found to decrease levels of vascular endothelial growth factor (VEGF), interleukin-6 (IL-6), IL-8, and tumor necrosis factor-α (TNF-α) in animal studies [18,19]. In mixed-density and chronic SDHs, these inflammatory and fibrinolytic factors are likely the cause of chronic capsular fragility and poor neovascularization [15]. VEGF levels are significantly increased within hematoma fluid and neomembranes compared with serum samples, and its presence is associated with increased levels of TNF-α and hypoxia-inducible factor-1 α (HIF-1α), both of which are known as VEGF inducers [20,21]. Although VEGF is normally a proangiogenic factor, the excessive presence of VEGF has been associated with pathological angiogenesis, resulting in leaky, friable vessels [22]. This is likely related to a VEGF-induced increase in angiopoietin-2 expression, which in turn upregulates placental growth factor (PlGF) expression [23,24]. Excessive PlGF activity has been related to pathological angiogenesis in diseases such as diabetic retinopathy and has been found at significantly increased levels in CSDH fluid compared with serum [22,24]. Hematoma fluid has also been found to contain a very low ratio of soluble VEGF receptor 1 (a VEGF antagonist) to PlGF, indicative of an environment conducive to pathological angiogenesis [22].

Impaired angiogenesis within the neomembrane may in turn predispose to leakage of blood from immature vessels, resulting in recurrence [17,25]. Evidence for this was presented by Ito et al., who administered chromium-labeled red cells to patients with CSDH and measured the amount of labeled red cells found in the hematoma fluid upon aspiration 24 hours later [26]. They found that approximately 10% of the aspirate was made up of labeled red cells, which had accumulated in the hematoma over the 24-hour period. The rationale behind statin usage improving rates of recurrence is therefore clear: statins decrease levels of inflammatory factors that normally induce pathological angiogenesis within the hematoma neomembrane, resulting in decreased pathological angiogenesis and chronic leakage. In our cohort, only five of 89 hematomas in statin users experienced a recurrence, yielding a significantly lower rate of recurrence compared with nonusers, and an odds ratio of 0.22 (95% CI 0.08, 0.6; p=0.004). This suggests that statin use may be protective against recurrence in subacute and mixed-density SDHs and underscores the potential value of statins as an adjuvant medical treatment to be administered to patients who undergo surgical intervention for nonacute SDHs.

Antiplatelet and anticoagulant agents, on the other hand, have been inconsistently reported to increase the risk of recurrence. Kim et al. [5] found the two to be significantly associated with greater recurrence rates, but Torihashi et al. [9] and Mori and Maeda [3] reported no significant association. Several recent meta-analyses and reviews have assessed both the rates of recurrence in patients on antiplatelet/anticoagulant agents and the proper timing of resumption of said therapies. The findings were mixed, but with a greater number of studies demonstrating no significant difference in recurrence rates among antiplatelet/anticoagulant users than those that did [27, 28]. Additionally, there was no significant difference in rebleed rates among patients restarting antiplatelet/anticoagulant agents early in the postoperative period compared with those in which they were restarted late [29]. In our cohort, antiplatelet and anticoagulant users did not have a significantly different rate of recurrence when compared with nonusers. Thus, our results support the findings of the majority of studies that recurrence rates of nonacute SDHs are not increased by the use of antiplatelet or anticoagulant medications.

Mixed-density hematoma subtype was associated with significantly longer LOS and ICU LOS on multivariate analysis. Additionally, cisternal effacement, hematoma density, change in thickness of SDH after surgical intervention, age, hematoma subtype, and presenting GCS were all associated with significantly different discharge dispositions. Greater preoperative thickness was the only factor associated with significantly greater mortality. This would suggest that although these factors did not necessarily influence recurrence rate, they did significantly impact patient outcomes in terms of postoperative morbidity and mortality.

Limitations

The principal limitation of this study was its retrospective nature. As a result, there was no standardization in terms of surgical procedures or post-treatment management, including duration of drain placement, follow-up, readmission protocols, or thresholds for reoperation. Additionally, the decision to start statin therapy was at the discretion of the attending neurosurgeon and was done independently of any studies or trials. This variation in the treatment paradigm could result in a confounding effect on the results. Another limitation was the sample size, particularly that of recurrent hematomas requiring reoperation. Although the total number of cases was 268, only 41 recurred. Additionally, reoperation is an inherently difficult complication to assess, as it may occur very remotely from the initial hospital stay and may not be reported to the same institution. These findings would therefore benefit from future, large-scale, multicenter prospective work to reinforce the results. Finally, studies incorporating other adjuvant therapies such as middle meningeal artery embolization, which has been shown to reduce recurrence in chronic SDH [30], may help develop a new treatment paradigm for patients with this disease.

## Conclusions

This single-institution experience suggests that, in elderly patients, statin usage may provide a significant benefit in the reduction of recurrence requiring reoperation, whereas premorbid antiplatelet and anticoagulant medication usage did not alter reoperation rates significantly. Mixed-density SDHs were the most common subtype in which subsequent reoperation was required, and death occurred most commonly in this group as well. Hematoma subtype was also associated with significantly different ICU and hospital LOS. Although hematoma size did not correlate with significantly different reoperation rates, it was associated with significantly worse discharge disposition and was the only factor to correlate significantly with mortality. Further prospective studies and randomized controlled trials would help qualify the findings of the inquiry.
